# Comparison of antibiotic dosing recommendations for neonatal sepsis from established reference sources

**DOI:** 10.1007/s11096-018-0589-9

**Published:** 2018-01-16

**Authors:** T. B. Y. Liem, E. M. A. Slob, J. U. M. Termote, T. F. W. Wolfs, A. C. G. Egberts, C. M. A. Rademaker

**Affiliations:** 1 Department of Clinical Pharmacy, Wilhelmina Children’s Hospital, University Medical Centre Utrecht, Lundlaan 6, 3584 EA Utrecht, The Netherlands; 2 Division of Pharmacoepidemiology and Clinical Pharmacology, Faculty of Science, Utrecht University, Universiteitsweg 99, 3584 CG Utrecht, The Netherlands; 3 Department of Neonatology, Wilhelmina Children’s Hospital, University Medical Centre, Lundlaan 6, 3584 EA Utrecht, The Netherlands; 4 Department of Pediatrics, Infectious Diseases and Immunology, Wilhelmina Children’s Hospital, Lundlaan 6, 3584 EA Utrecht, The Netherlands

**Keywords:** Antibiotics, Dosing recommendations, Dosing variation, Neonatal sepsis, Neonates

## Abstract

*Background* Incorrect dosing is the most frequent prescribing error in neonatology, with antibiotics being the most frequently prescribed medicines. Computer physician order entry and clinical decision support systems can create consistency contributing to a reduction of medication errors. Although evidence-based dosing recommendations should be included in such systems, the evidence is not always available and subsequently, dosing recommendations mentioned in guidelines and textbooks are often based on expert opinion. *Objective* To compare dosage recommendations for antibiotics in neonates with sepsis provided by eight commonly used and well-established international reference sources. *Setting* An expert team from our Dutch tertiary care neonatal intensive care unit selected eight well-established international reference sources. *Method* Daily doses of the seven most frequently used antibiotics in the treatment of neonatal sepsis, classified by categories for birth weight and gestational age, were identified from eight well-respected reference sources in neonatology/pediatric infectious diseases. *Main outcome measure* Standardized average daily dosage. *Results* A substantial variation in dosage recommendations of antibiotics for neonatal sepsis between the reference sources was shown. Dosage recommendations of ampicillin, ceftazidime, meropenem and vancomycin varied more than recommendations for benzylpenicillin, cefotaxime and gentamicin. One reference source showed a larger variation in dosage recommendations in comparison to the average recommended daily dosage, compared to the other reference sources. *Conclusion* Antibiotic dosage recommendations for neonates with sepsis can be derived from important reference sources and guidelines. Further exploration to overcome variation in dosage recommendations is necessary to obtain standardized dosage regimens.

## Impacts on Practice

- There appears to be a significant variability of neonatal dosing recommendations for antibiotics for neonatal sepsis between established reference sources.
- It is important to attain uniformity in neonatal dosage recommendations of antibiotics. Expert committees should take a lead in interpreting the existing evidence and in establishing uniform dosage recommendations.
- To provide children with effective and safe medicines, knowledge based formularies should be developed.

## Introduction

The most common medication error in neonates is incorrect dosing due to lack of evidence or lack of access to the available evidence at the moment of prescribing [1]. Computer physician order entry (CPOE) and clinical decision support systems can contribute to the reduction of such medication errors and thereby increase patient safety [2]. To obtain full benefit of these systems, evidence-based dosing recommendations should be included, which unfortunately are not always available for neonates because of the lack of pharmacokinetic data and clinical efficacy studies in this vulnerable patient group [3]. As a consequence, dosing in neonates is often based on clinical experience and expert-opinion. This is probably one of the reasons for high variability in dosing of frequently used antibiotics in European neonatal intensive care units (NICUs) [3].

Moreover, a multicentre study on paediatric antimicrobial prescribing in European hospitals demonstrated that the prescribed daily dose (PDD) in children increased with age and weight. This advocates the need to define standardized paediatric daily doses for different paediatric age groups and neonates separately [4]. In this perspective, a first step towards uniformity in neonatal antibiotic dosage recommendations was previously taken by us through the development of a set of neonatal defined daily doses (nDDDs) [5].

Among the drugs most frequently used in NICUs antimicrobial agents rank highest [6], since the multiple risk factors for infection in preterm immunocompromised infants result in a low threshold for the initiation of antimicrobial therapy. Neonatal infections, predominantly sepsis, are a significant cause of morbidity and mortality in the newborn, particularly in preterm, low birth weight infants [7].

### Aim of the study

The aim of this study is to compare the dosage recommendations for commonly used antibiotics in neonatal sepsis from eight frequently used and well-established international neonatal/paediatric reference sources.

### Ethics approval

This article does not contain any studies with human participants or animals performed by any of the authors.

## Method

### Selection of antibiotics and reference sources

In this study the focus was on the variation in dosage recommendations of antibiotics for neonatal sepsis. Based on a survey on antibiotic use in all ten Dutch tertiary care neonatal intensive care units (NICUs) [8], the ten most frequently used antibiotics in neonates in the Netherlands were selected: ampicillin, amoxicillin, amoxicillin–clavulanic acid, benzylpenicillin, flucloxacillin, ceftazidime, cefotaxime, meropenem, gentamicin and vancomycin.

A team of experts from our children’s hospital, including a paediatric-infectious disease specialist, a neonatologist and several hospital pharmacists, selected nine commonly used and well-established international references in neonatology/paediatrics and paediatric infectious diseases to be evaluated for dosage recommendations [5], namely: four general paediatric dosage handbooks, four neonatal/paediatric infectious diseases handbooks and one paediatric online formulary:

- Dutch Paediatric Formulary (DPF) (Dutch Expertise Network on Paediatric Pharmacotherapy, NKFK), online accessible [9].
- Infectious Diseases of the Fetus and the Newborn Infant (Remington & Klein), 8th edition, 2016 [10].
- Micromedex Neofax Online (Neofax) [11].
- The Harriet Lane Handbook, 20th edition, 2015 [12].
- Red Book, 2015 [13].
- Principles and Practices of Paediatric Infectious Diseases (Long & Pickering), 4th edition, 2012 [14].
- Nelson’s Pocket Book of Paediatric Antimicrobial Therapy (Nelson’s), 22nd edition, 2016 [15].
- Pediatric & Neonatal Dosage Handbook (PDH), 22th edition, 2015 [16].
- The British National Formulary for children, 2015–2016 (BNFC) [17].

### Inclusion criteria

As a primary condition for comparing dosage recommendations, at least four reference sources had to provide dosage recommendations for the specific antibiotic agent.

### Exclusion criteria

The reference sources referring to other guidance documents, which were not based on primary literature sources, were excluded.

### Determination of dosage recommendations

All intravenous dosage recommendations [recommended daily dosage (RDD)] for neonates with sepsis for the selected antibiotics mentioned in the included reference sources were collected, as well as any referenced evidence referring to original clinical studies in neonates.

In addition, to be able to compare the dosage recommendations, these were converted to the format ‘mg/kg/day in x divided doses’, if possible. To avoid interpretation errors, age categories were unambiguously compared, i.e., dosage recommendations for different age-categories were excluded. In case of a dosage recommendation with a dosage range as well as an interval range (e.g., 10–20 mg/kg/day every 6–8 h) the limits were mediated as RDD. In the aforementioned example, the daily dose limits would be 30–80 mg/kg/day and the RDD would be 55 mg/kg/day.

Next, for each antibiotic and age category the average of the dosage recommendations in the eight reference sources was calculated and expressed as the aRDD. Subsequently, to evaluate similarities and differences between the dosage recommendations and the aRDD, the deviation of each RDD relative to the aRDD was calculated. The calculation of the deviation was determined by the formula: deviation (%) = − (100 − (RDD/aRDD *100%)). In this formula, the aRDD was seen as 100%.

## Results

### Characteristics of reference sources

The dosage recommendations of Remington & Klein were based on those in the Red Book and Neofax. Since these two latter reference sources were already included in our comparison, we did not include Remington & Klein for further analysis.

The BNFC, Neofax and PDH were the only reference sources that included dosage recommendations for almost all ten selected antimicrobial agents (nine out of ten). Table 1 shows the characteristics of the eight analysed reference sources. The Red Book, Nelson’s and Long & Pickering did not include age-dependent categories for dosage recommendations. The Dutch Paediatric Formulary, Harriet and Lane handbook and Nelson’s were based on indications.

**Table 1:** Characteristics of eight established reference sources in paediatrics and paediatric infectious diseases

Characteristics	DPF [9]	Neofax [11]	The Harriet Lane Handbook [12]	Red Book [13]	Long & Pickering [14]	Nelson’s [15]	PDH [16]	BNFC [17]
Recommendations for neonates available	Yes	Yes	Yes	Yes	Yes	Yes	Yes	Yes
Recommendations for preterms available	Yes	Yes	Yes	No	No	Yes	No	Yes
Approach based on indication	Yes	No	Yes	No	No	Yes	No	No
Approach based on antibiotic	Yes	Yes	Yes	Yes	Yes	Yes	Yes	Yes
Detailed age and weight categorisation available	Yes	Yes	Yes	No	No	No	Yes	Yes
Literature references mentioned	No	Yes	No	No	Yes	No	Yes	No
Mean difference from the aRDD (%)	15.8	23.1	24.6	15.3	46.4	23.3	25.2	17.4

All reference sources included *neonatal* dosage recommendations. Most of the reference sources had dosage recommendations for *preterm infants,* but the Red Book, Long & Pickering and PDH did not. However, all reference sources used different age categories for both populations based on birth or current weight, gestational age (GA) or postmenstrual age (PMA).

All reference sources used literature referencing for their dosage recommendations. However, only three reference sources mentioned their literature referencing, i.e., the PDH, Long & Pickering and the Neofax. The DPF mentioned the literature references partially, no references were described for ceftazidime and vancomycin. The Neofax referred to the Red Book 2009 for the dosage recommendations of ampicillin and penicillin G. The Red Book 2015 was a literature reference for dosage recommendations of gentamicin, ampicillin, ceftazidime, benzylpenicillin and cefotaxime in the PDH. The highest total number of literature references for each antibiotic agent was in the Neofax, of which meropenem had 16 references. Cefotaxime and ceftazidime had the lowest number of references. Comparison of all available references illustrated that only a few were cited in common.

### Dosage recommendations

In total 309 dosage recommendations were included for comparison. Flucloxacillin, amoxicillin and amoxicillin-clavulanic acid were excluded because less than four reference sources included dosage recommendations for these agents. Therefore, seven of the initial ten selected antibiotics were evaluated. Table 2 illustrates the variation in dosage recommendations between the reference sources for the seven analysed antibiotics.

**Table 2:** Comparison of dosage recommendations of seven most commonly used antibiotics for sepsis in neonates in eight reference sources

	DPF [9]	Neofax [11]	The Harriet Lane Handbook [12]	Red Book [13]	Long & Pickering [14]	Nelson’s [15]	PDH [16]	BNFC [17]	aRDD	Range
Ampicillin (mg^−1^ kg^−1^ day^−1^)										
< 7 days, ≤ 2000 g	n.a.^a^	50–150	50–100	100	200	100	100	60–120	101	50–200
< 7 days, > 2000 g	n.a.^a^	50–150	75–150	150	200	150	150	60–120	132	50–200
≥ 1 week, ≤ 1200 g	n.a.^a^	50–150	50–100	150	300	150	150	90–240	146	50–300
≥ 1 week, 1200–2000 g	n.a.^a^	50–150	75–150	150	300	150	100–150	90–240	151	50–300
≥ 1 week, ≥ 2000 g	n.a.^a^	50–150	100–200	200	300	150	150–200	90–240	180	50–300
Benzylpenicillin/Penicillin G (IU^−1^ kg^−1^ day^−1^)										
< 7 days, ≤ 2000 g	50,000	50,000–150,000	50,000–100,000	50,000–100,000	n.a.	100,000	50,000–100,000	100,000	82,143	50,000–100,000
< 7 days, > 2000 g	75,000	50,000–150,000	75,000–150,000	50,000–100,000	n.a.	100,000	50,000–100,000	100,000	90,357	75,000–150,000
≥ 1 week, ≤ 1200 g	75,000	50,000–150,000	50,000–100,000	100,000–200,000	n.a.	150,000	50,000–100,000	100,000	100,000	50,000–225,000
≥ 1 week, 1200–2000 g	75,000	50,000–150,000	75,000–150,000	100,000–200,000	n.a.	150,000	50,000–100,000	120,000	111,071	50,000–225,000
≥ 1 week, ≥ 2000 g	100,000	50,000–150,000	100,000–200,000	100,000–200,000	n.a.	150,000	50,000–100,000	120,000	120,714	50,000–200,000
Cefotaxime (mg^−1^ kg^−1^ day^−1^)										
< 7 days, ≤ 2000 g	100	100–150	100	100	100	100	100	50–100	100	50–200
< 7 days, > 2000 g	100	100–150	100–150	100	100	100	100–150	50–100	106	50–200
≥ 1 week, ≤ 1200 g	150	100–150	100	100–150	150	150	100–150	75–150	123	75–200
≥ 1 week, 1200–2000 g	150	100–150	150	100–150	150	150	100–150	75–150	135	75–200
≥ 1 week, ≥ 2000 g	150	100–150	150–200	200	150	150	150–200	75–150	148	75–200
Ceftazidime (mg^−1^ kg^−1^ day^−1^)										
< 7 days, ≤ 2000 g	50	60–90	100	100	n.a.	100	25–100	25–50	82	25–100
< 7 days, > 2000 g	100	60–90	100–150	100	n.a.	100	100	25–50	91	25–100
≥ 1 week, ≤ 1200 g	100	60–90	100	100–150	n.a.	150	100–150	75–150	113	75–150
≥ 1 week, 1200–2000 g	100	60–90	150	100–150	n.a.	150	100–150	75–150	127	75–150
≥ 1 week, ≥ 2000 g	150	60–90	150	200	n.a.	150	150	75–150	141	75–200
Gentamicin (dose to start, mg^−1^ kg^−1^ day^−1^)										
< 7 days, ≤ 2000 g	2.5–3.3	3.0	2.0–5.0	2.5	4.0	2.5	2.5	3.3	3.0	2.0–5.0
< 7 days, > 2000 g	2.5–3.3	3.0	2.0–5.0	4.0	4.0	2.5	4	3.3	3.6	2.5–5.0
≥ 1 week, ≤ 1200 g	4.0	3.2	4.0	3.3	4.0	2.5–5.0	3.3	5.0	3.7	2.2–8.0
≥ 1 week, 1200–2000 g	4.0	3.2	4.0	3.3	4.0	2.5–5.0	3.3	5.0	4.3	1.9–8.0
≥ 1 week, ≥ 2000 g	4.0	3.2	4.0	4.5	4.0	2.5–5.0	4.5	5.0	4.7	1.9–8.0
Meropenem (mg^−1^ kg^−1^ day^−1^)										
< 7 days, ≤ 2000 g	40	60	20–30	40	n.a.	40	40	40	41	20–60
< 7 days, > 2000 g	40	60	20–30	60	n.a.	60	60	40	46	20–60
≥ 1 week, ≤ 1200 g	60	90	20–30	60	n.a.	60–90		60	59	20–90
≥ 1 week, 1200–2000 g	60	90	20–30	60	n.a.	60–90	40	60	54	20–90
≥ 1 week, ≥ 2000 g	60	90	20–30	60–90	n.a.	60–90	60–90	60	64	20–90
Vancomycin (mg^−1^ kg^−1^ day^−1^)										
< 7 days, ≤ 2000 g	20	13.3–30	25	b.scr.	45	b.scr.	20	22.5	24	15–45
< 7 days, > 2000 g	20	13.3–30	25	b.scr.	45	b.scr.	30	22.5	27	15–45
≥ 1 week, ≤ 1200 g	30	13.3–30	17.5	b.scr.	45	b.scr.	15	22.5	24	15–45
≥ 1 week, 1200–2000 g	30	13.3–30	17.5	b.scr.	45	b.scr.	30	22.5	27	15–45
≥ 1 week, ≥ 2000 g	48	13.3–30	17.5	b.scr.	45	b.scr.	40–45	22.5	30	15–60

*DPF* Dutch Paediatric Formulary, *BNFC* The British National Formulary for children, *aRDD* average recommended daily dosage, *mg* milligrams, *kg* kilograms, *IU* international units, *n.a.* not available, *b.scr.* based on serum creatinine

^a^Instead of ampicillin, amoxicillin is used in the Netherlands. The DPF provides amoxicillin dose recommendations

**Fig. 1:**
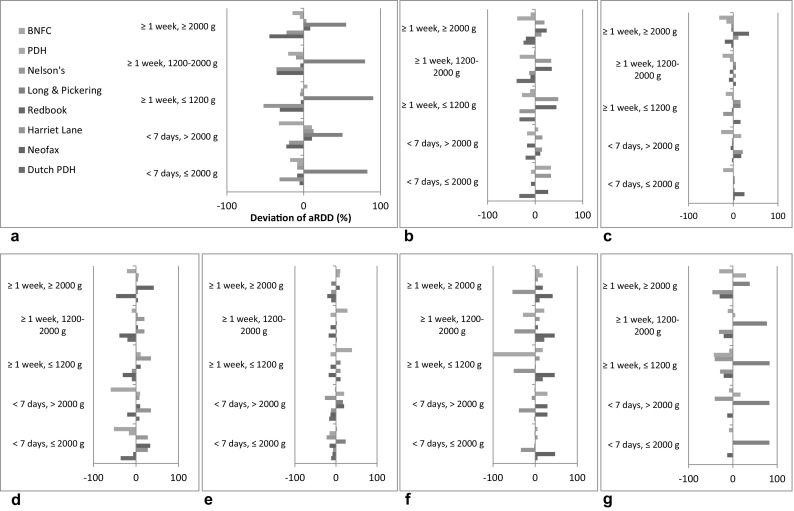
Variation in standardized dosage recommendations for antibiotics in eight commonly used and well-established reference sources. **a** Ampicillin. **b** Benzylpenicillin/Penicillin G. **c** Cefotaxime. **d** Ceftazidime. **e** Gentamicin. **f** Meropenem. **g** Vancomycin

Figure 1 shows the variation in standardized dosage recommendations in comparison to the aRDD for each evaluated reference source. The relative deviation of the RDD compared to the aRDD is shown for each antibiotic and reference source. Between the evaluated reference sources the relative deviation of the RDD compared to the aRDD for ampicillin, ceftazidime, meropenem and vancomycin is above 50%, in contrast to benzylpenicillin, cefotaxime and gentamicin.

Long & Pickering showed larger variation in dosage recommendations in comparison to the aRDD, compared to the other reference sources. On the other hand, the BNFC demonstrated the least variation in dosage recommendations in comparison to the aRDD of all evaluated reference sources.

## Discussion

To our knowledge, this is the first study that reviewed eight internationally well-respected reference sources for dosage recommendations of antibiotics for neonatal sepsis and showed a substantial variation between reference sources therein. The dosing recommendations of ampicillin, ceftazidime, meropenem and vancomycin showed a larger variation compared to those of benzylpenicillin, cefotaxime and gentamicin.

The Summaries of Product Characteristics (SmPCs) of the concerning antibiotics were evaluated when specific dosage recommendations for neonatal sepsis were available. These neonatal dosage recommendations were explicitly available in the SmPC of cefotaxime and gentamicin exclusively, which might be an explanation for the smaller variation in dosage recommendations between the evaluated reference sources for these two antibiotics compared to ampicillin, ceftazidime, meropenem and vancomycin.

Furthermore, in comparison with the other seven evaluated reference sources Long & Pickering demonstrated a larger variation in its dosage recommendations in comparison to the aRDD. It is, however, difficult to explain why this reference source stands out in the comparison of dosage recommendations. One possible explanation for this discrepancy could be that Long & Pickering has a focus on diagnosis and management of paediatric infectious diseases rather than providing a complete neonatal antibiotic dosage recommendation.

Overall, there may be several reasons for the considerable variation between some antibiotic dosage recommendations. First of all, the lack of clinical efficacy studies in the neonatal population [3, 18]. Furthermore, large trials are required to show any differences in clinical efficacy between dosage recommendations, which are hard to set up in this vulnerable population in practical and ethical perspective, let alone excellent pharmacokinetic (PK)-pharmacodynamic (PD) studies including appropriate biomarkers as endpoint. Over the last few years some new PK-PD studies have been carried out [19–21], but only a few studies published before 2010 are included as a literature reference in the investigated reference sources. Second, due to geographical regional variation in antimicrobial susceptibility patterns, empirical therapy should be guided by local susceptibility patterns resulting in variation in dosage recommendations [22]. Third, problems in adoption and dissemination of evidence based knowledge can cause high dosing variability [3]. For example, the BNFC recommended to double the dose in severe infections/meningitis. This might be an explanation for some differences in dosing recommendations. Finally, one could hypothesize that the variation might be explained by the differences in the procedure of establishing the dosing recommendations between the reference sources, e.g., composition of editorial board, availability of references or frequency of updating. Regarding the latter, an additional remarkable finding was that five out of eight reference sources evaluated in our study were paper ones. In our opinion, the era of using reference sources in book form has come to an end. One should henceforth give higher preference to available *online* (electronic) information of antibiotic dosage recommendations as these can be updated regularly.

The variation between recommendations from different sources was also seen recently by systematically comparing different sources of drug information regarding dose adjustment for renal function [23] and those on safety in lactation [24]. These inconsistencies in reference sources, guidelines and drug management programmes might not encourage adherence to the recommendations from these sources and subsequently might lead to more *experience*-based instead of *evidence*-based medicine [25].

This study had the aim to map differences in dosage recommendations. It was therefore not intended as a qualitative judgement about the appropriateness of dosage recommendations nor intended to judge the quality of the reference sources.

Inconsistencies in neonatal dosage recommendations for antibiotics from these common reference sources might indirectly contribute to the difficulty in clinical practice in determining an appropriate neonatal dosage [26]. Hence, standardization of neonatal antibiotic dosing schemes is desirable, which potentially may lead to better outcome and less toxicity. Moreover, a standardized dosing regimen and therapeutic drug monitoring (TDM) would help reduce medication errors as the National Patient Safety Agency (NPSA) report (Review of Patient Safety for Children and Young People, June 2009) concluded [27]. However, it is generally known from several recent PK-PD studies that routine TDM for exclusively gentamicin and vancomycin in neonates was strongly recommended in contrast to penicillins and cephalosporins, since both antibiotics have a small therapeutic window and overdosing can lead to severe toxicity [19, 21, 28, 29].

Uniformity in neonatal dosage recommendations of antibiotics should be achieved and also evidence based. Evidence should be derived from established international reference sources, guidelines and corresponding SmPCs. In addition, not only prospective validation of neonatal dosing regimens of antibiotics, but also further exploration of pharmacokinetic and pharmacodynamic aspects of antibiotics in neonates is therefore essential [4].

Expert committees should take a lead in interpreting the existing evidence and in establishing uniform dosage recommendations, adopting the Grading of Recommendations Assessment, Development and Evaluation (GRADE) system preferably [30]. In this context, the development and implementation of a national knowledge-based formulary for children in the Netherlands may serve as an successful example [31]. International consensus is required to harmonize the way existing data is presented and to develop better dosage regimens. Ideally, dosage recommendations for neonatal sepsis should be included in the SmPCs in case these are not mentioned herein.

## Conclusion

Our comparison of dosage recommendations in eight internationally well-respected reference sources led to the conclusion that the dosage recommendations for ampicillin, ceftazidime, meropenem and vancomycin for neonatal sepsis varied considerably. Further exploration to overcome variation in dosage recommendations is necessary to obtain established dosage regimens and thus full benefit of CPOE and clinical decision support systems in neonatology.
